# The Association of Annexin A1 and Chemosensitivity to Osimertinib in Lung Cancer Cells

**DOI:** 10.3390/cancers13164106

**Published:** 2021-08-15

**Authors:** Min-Chun Chuang, Jr-Hau Lung, Yi-Chuan Chen, Yu-Ching Lin, Ya-Chin Li, Ming-Szu Hung

**Affiliations:** 1 Department of Pulmonary and Critical Care Medicine, Chang Gung Memorial Hospital, Chiayi 61363, Taiwan; ma7390@hotmail.com (M.-C.C.); lin0927@cgmh.org.tw (Y.-C.L.); c39958@yahoo.com.tw (Y.-C.L.); 2 Department of Nursing, Chang Gung University of Science and Technology, Chiayi Campus, Chiayi 61363, Taiwan; 3 Department of Medical Research, Chang Gung Memorial Hospital, Chiayi 61363, Taiwan; jrhaulung@gmail.com; 4 Department of Emergency Medicine, Madou Sin-Lau Hospital, The Presbyterian Church in Taiwan, Tainan 72100, Taiwan; giomacky@gmail.com; 5 Department of Medicine, College of Medicine, Chang Gung University, Taoyuan 33323, Taiwan; 6 Department of Respiratory Care, Chang Gung University of Science and Technology, Chiayi Campus, Chiayi 61363, Taiwan

**Keywords:** lung cancer, annexin A1, target therapy

## Abstract

**Simple Summary:**

Annexin A1 (ANXA1) is associated with the growth and resistance to chemotherapy drugs in lung cancer cells. In this study, the association of ANXA1 with chemosensitivity to Osimertinib, a third generation epidermal growth factor receptor tyrosine kinase inhibitor (EGFR-TKI) was studied. The knockdown of ANXA1 increased chemosensitivity to Osimertinib and decreased tumorigenesis, invasion and migration of lung cancer cells with EGFR mutations. The study showed that ANXA1 plays critical roles in chemosensitivity to Osimertinib in lung cancer cells with EGFR mutations.

**Abstract:**

Annexin A1 (ANXA1) has been reported to promote tumor growth and resistance to chemotherapy drugs in lung cancer cells. In this study, we focused on the association of ANXA1 and chemosensitivity with a third generation epidermal growth factor receptor-tyrosine kinase inhibitor (EGFR-TKI), Osimertinib, in lung cancer cells with EGFR mutations. The overexpression of ANXA1 was observed in the lung cancer cells studied. The downregulation of ANXA1 with small interference RNA (siRNA) decreased the growth of lung cancer cells. In lung cancer cells with EGFR mutations, the knockdown of ANXA1 increased the chemosensitivity to Osimertinib, and decreased the tumorigenesis, invasion and migration of lung cancer cells. Further study showed that the knockdown of ANXA1 inhibited the phosphorylation of EGFR and down-stream Akt pathways and promoted apoptosis in lung cancer cells treated with Osimertinib. A mice xenograft lung cancer model was established in our study and showed that ANXA1 siRNA enhanced the effects of Osimertinib in vivo. Our study results showed that ANXA1 plays critical roles in chemosensitivity to EGFR-TKI in lung cancer cells with the EGFR mutation. Our efforts may be used in the development of lung cancer treatment strategies in the future.

## 1. Introduction

Lung cancer remains the leading cause of cancer deaths in the world. At the time of diagnosis, most lung patients are in the advanced stage of the disease. The prognosis of advanced lung cancer has not been satisfactory, despite aggressive treatment [1], and developing new treatment strategies for lung cancer is necessary at present. For instance, the discovery of tyrosine kinase inhibitors targeting mutant epidermal growth factor receptor (EGFR) has provided a good example of lung cancer target therapy [2]. EGFR signaling is activated by the binding of EGF-like growth factors, leading to the dimerization of adjacent EGFR molecules or hetero-dimerization with other EGFR families. Then, the subsequent transphosphorylation of the intracellular tyrosine kinase domains initiates a transduction of intracellular signals, and leads to the proliferation, angiogenesis, invasion and metastasis of lung cancer [3]. In non-small cell lung cancer (NSCLC), the EGFR tyrosine kinase (TK) domain mutations in cancer cells are strongly associated with a higher sensitivity to EGFR tyrosine kinase inhibitor (TKI). The presence of EGFR mutations varies in different races, which is around 10% in NSCLC patients in Europe and North America, and 30–40% in East Asia. EGFR mutations in NSCLC are usually present on exons 18–21 of the *EGFR* gene. The missense mutations in exon 21 (L858R), and in-frame deletions within exon 19 (delE746-A750) are common EGFR mutations [4]. In NSCLC patients, previous studies have proved that these mutations are associated with a favorable response to first-line treatment with EGFR-TKIs, such as gefitinib, erlotinib and afatinib, compared to standard chemotherapy in NSCLC [5,6,7]. However, even with the activating mutation, patients developed resistance to EGFR-TKI after a certain period of treatment. The most common secondary mutation, detected in approximately 60% of the acquired resistance developed to first-line EGFR-TKIs, is the T790M mutation in exon 20 of the *EGFR* gene [8]. At present, the T790M resistance can be overcome by using third-generation EGFR tyrosine kinase inhibitors, such as Osimertinib. The T790M mutation-positive advanced NSCLC initially responding to Osimertinib usually develops resistance to treatment, with a progression-free survival ranging from 9.7 to 11 months [9]. As a result, it is necessary to develop novel treatments to overcome Osimertinib resistance in the present era.

Annexin A1 (ANXA1) is a 37 kDa protein with calcium and phospholipid binding properties [10]. ANXA1 is also known as lipocortin I and is encoded by the *ANXA1* gene located on chromosome 19q24. ANXA1 has its critical effects on proliferation, membrane aggregation, inflammation, cellular transduction, phagocytosis, differentiation and apoptosis [11]. ANXA1 has been reported to promote the development of tumorigenesis in lung cancer cells [12]. The overexpression of ANXA1 was observed in NSCLC cells and tissues. ANXA1 knockdown suppresses the proliferation, migration and invasion of NSCLC cells [12]. The overexpression of ANXA1 is also associated with resistance to chemotherapy drug cisplatin in NSCLC cells [13]. The upregulation of ANXA1 was also observed in lung cancer tissues compared to normal lung tissues and high levels of serum ANXA1 was associated with an advanced pathological grade and stage in lung cancer patients [14]. The above findings imply that ANXA1 contributes to the growth and invasion of NSCLC cells, and ANXA1 may be a promising target for the development of the treatment of NSCLC. Moreover, ANXA1 also plays a critical role in the EGFR pathway. ANXA1 is also reported to act directly on the EGFR and regulate the EGFR/Ras pathway. Through binding to the EGFR adaptor protein Grb2, ANXA1 increases Ras activity and then results in the activation of the mitogen-activated protein kinase extracellular signal-regulated kinase [15].

To date, the association of ANXA1 with chemosensitivity to EGFR-TKI has not been described. In this study, we focused on the association of ANXA1 and chemosensitivity to Osimertinib in NSCLC lung cancer cells with EGFR mutations.

## 2. Materials and Methods

### 2.1. Cell Culture

NSCLC cell lines A549 (CCL-185), H1650 (CRL-5883), H1975 (CRL-5908), H460 (HTB-177), H157 (CRL-5802), H1703 (CRL-5889) and a fetal lung fibroblast cell line WI-38 were bought from American Type Culture Collection (Manassas, VA, USA). The PC9 cell line was obtained from National Taiwan University as a generous gift from Professor Pan-Chyr Yang. Lung cancer cells were cultured in RPMI-1640 complete growth medium. The medium was supplemented with 10% fetal bovine serum (FBS), 10 units/mL penicillin and 10 µg/mL streptomycin and incubated at 37 °C and 5% CO_2_.

### 2.2. Small Interfering RNA (siRNA) Transfection

Non-target siRNA (5′-UGGUUUACAUGUCGACUAA-3′) and pre-designed and validated ANXA1 siRNAs (5′-AUUCUAUCAGAAGAUGUAU-3′, 5′-CAAAGGUGGUCCCGGAUCA-3′, 5′-GAAGUGCGCCACAAGCAAA-3′) and a universal non-target siRNA (5′-UGGUUUACAUGUCGACUAA-3′) were purchased from Dharmacon (Lafayette, LA, USA) and used for further studies. Transfection was performed using Lipofectamine^TM^ RNAiMAX Transfection Reagent (Invitrogen, Carlsbad, CA, USA) according to the manufacturer’s manual.

### 2.3. Cell Viability Assay

Cell viability assay after ANXA1 siRNA or Osimertinib treatment was performed. First, lung cancer cells (2 to 4 × 10^3^) were plated in 96-well plates in antibiotic-free media. When the growth of cells reached 80% confluence, transfection was performed with a final concentration of 50 nM for each siRNA. After transfection, cells were then treated with the indicated concentration of Osimertinib for the specified hours. Viable cells were analyzed using CellTiter-Glo luminescent cell viability assay (Promega, Madison, WI, USA), and the luminescent signal was read by EnSpire^®^ Multimode Plate Reader (PerkinElmer, Waltham, MA, USA).

### 2.4. Colony Formation Assay

For the anchorage-dependent colony formation assay, H1975 lung cancer cells were transfected with 50 nM ANXA1 siRNA. After transfection for 48 h, cells (5 × 10^2^) were plated in 10 cm culture dishes. Cells were then incubated in complete medium with or without 10 nM Osimertinib. After 14 days, the colonies were fixed with 10% (*v*/*v*) methanol and stained with 0.1% crystal violet. Colonies with more than 50 cells were counted.

### 2.5. Three-Dimensional Culture

Three-dimensional culture was used for anchorage-independent colony formation. The H1975 lung cancer cells (2 × 10^4^) were cultured in Corning™ 96-Well Clear Ultra Low Attachment Microplates (Thermo Fisher Scientific). Three-dimensional culture medium was prepared using an FCeM™-series Preparation kit (Nisan Chemical, Tokyo, Japan). Cells were transfected with 50 nM non-target or ANXA1 siRNA and treated with or without 10 nM Osimertinib. After 10 days, colonies were photographed and counted.

### 2.6. Wound Healing Assay

The wound healing assay was used to evaluate the ability of cell migration. H1975 lung cancer cells (5 × 10^5^) were cultured in a culture-insert (ibidi GmbH, Martinsried, Germany). After transfection with 50 nM non-target or ANXA1 siRNA, cells were treated with or without 10 nM Osimertinib for 24 h. The insert was then removed, and cells were cultured in pre-warmed fresh media. The gap was observed for haling status, photographed and measured at indicated time points.

### 2.7. Invasion Assay

The invasion assay was used to evaluate the ability for cell invasion. H1975 lung cancer cells were cultured in 24-well 6.5-millimeter diameter inserts (Corning, 8.0 mm pore size), which is coated with an indicator layer of growth factor reduced Matrigel (BD Transduction Laboratories). After transfection with non-target or ANXA1 siRNA, cells were treated with or without 10 nM Osimertinib. Cells were cultured in the upper well and incubated with 5% FBS and 100 ng/mL fibronectin in the lower chambers. Then, a cotton swab was used to remove cells in the upper chamber after 24 h. Cells that have migrated into the lower chamber were fixed in 4% paraformaldehyde and stained with 0.5% crystal violet. Filters were photographed, and the total number of cells on the filters was then quantified.

### 2.8. Western Bot Analysis 

The expression of protein was analyzed using Western blot analysis. Whole protein from cells was extracted using M-PER Mammalian Protein Extraction Reagent, containing Phosphatase Inhibitor Cocktail Set II (Calbiochem, San Diego, CA, USA) and Complete Protease Inhibitor Cocktails (Roche, Lewes, UK) according to the manufacturer’s protocol. Proteins (50 μg) were separated on 7.5% gradient sodium dodecyl sulfate (SDS)–polyacrylamide gels and then transferred electrophoretically to Immobilon-P membranes (Millipore, Billerica, MA, USA). Primary antibodies, including ANXA1 (Cell Signaling Technology, Danvers, MA, USA), phospho EGFR (Tyr1068) (Cell Signaling Technology), EGFR (Santa Cruz, Dallas, TX, USA), Akt (Santa Cruz), phospho AKT (Ser473) (Cell Signaling Technology), phospho HER2 (Tyr877) (Cell Signaling Technology), HER2 (Cell Signaling Technology), cleaved-PARP (Cell Signaling Technology) and β-actin (Sigma, St. Louis, MO, USA) were used. After binding to the indicated secondary antibodies, the detection of the antigen–antibody complex was performed using an enhanced chemiluminescence (ECL) blotting analysis system (GE Healthcare Life Sciences, Piscataway, NJ). The band of Western blot analysis was then quantified using ImageJ software (v1.44m for Windows, National Institutes of Health, Bethesda, MD, USA).

### 2.9. Apoptosis Detection

The H1975 lung cancer cells were cultured in 6-centimeter cell culture dishes. After 24 h, cells were transfected with 50 nM control non-target siRNA, or ANXA1 siRNA, with or with or without 100 nM Osimertinib (Sigma-Aldrich, Saint Louis, MO, USA). Cells were trypsinized after 72 h. Apoptosis was detected using an FITC Annexin V Apoptosis detection Kit (BD Bioscience, Franklin Lakes, NJ, USA) according to the manufacturer’s protocol. Cells were analyzed by a flow cytometry using FACS Calibur (BD Bioscience) and Cellquest™ Pro software (BD Bioscience).

### 2.10. Mice Xenograft Model

A mice xenograft model was established to evaluate the effects of ANXA1 siRNA and Osimertinib in vivo. The Institutional Animal Care and Use Committee at Chang Gung Memorial Hospital, Chiayi, Taiwan, approved all protocols of the animal study (no. 2017121801). Female Balb/c athymic nude mice of 5–6 weeks old were used for the animal study. The mice were housed under specific pathogen-free conditions. To perform the xenograft study, flank areas of mice were injected with H1975 lung cancer cells (1 × 10^6^) mixed in 100 μL of serum-free RPMI-1640 medium and 20% matrigel (BD Biosciences, San Jose, CA). The mice were divided into four groups randomly when the mean size of the tumor reached approximately 50 mm^3^. Then, mice were injected with either non-target or ANXA1 siRNAs (Accell-siRNA, Dharmacon, Lafayette, CO, USA) intratumorally once a week for 3 weeks with or without Osimertinib; then, 5 mg/kg, 3 times a week for 3 weeks was given by oral gavage. The formula (L × W^2^/2), where L represents the largest tumor diameter and W represents the smallest tumor diameter, was used to calculate the tumor volume twice a week measured using a caliper. The mice were sacrificed by carbon dioxide euthanasia at 29 days after lung cancer cell injection, and tumors were excised for further studies.

### 2.11. Immuno-Histochemical (IHC) Staining

To detect the expression of ANXA1 and Ki-67 in mice xenografts, IHC staining was conducted. First, 4-micrometer sections were cut from formalin-fixed, paraffin-embedded tissues, and then deparaffinized with xylene and dehydrated using a gradient ethanol series. Citric acid (pH 6.0) at 97 °C for 30 min, followed by treatment with 3% hydrogen peroxide, was performed for antigen retrieval. Overnight incubation of the slides at 4 °C with ANXA1 (Santa Cruz, CA) or Ki-67 (Spring Bioscience, CA) antibodies was performed. IHC staining was quantified using the semi-quantitative immunoreactive score (IRS), as described previously [16]. The IRS was calculated by multiplying the percentage of the positively stained cells (0 = 0% of cells stained; 1 = less than 10% of cells stained; 2 = 11–50% of cells stained; 3 = 51–80% of cells stained; 4 = more than 81% of cells stained) by the staining intensity (0 = no staining; 1 = weak staining; 2 = moderate staining; 3 = strong staining).

### 2.12. Transfection of Plasmids

The transfection of pCMV6-AC-GFP and pCMV6-ANXA1-GFP plasmids (Origene, Rockville, MD, USA) into H1975 and H1650 lung cancer cells was performed by using OMNIfect^TM^ transfection reagent (Transomic Technologies, Huntsville, AL, USA) according to the manufacturer’s protocol. Seventy-two hours after transfection and treated with or without indicated concentrations of Osimertinib, the survival cells were determined using a CellTiter 96^®^ AQ_ueous_ One Solution Cell Proliferation Assay (Promega, Madison, WI, USA) on a 96-well microplate reader.

### 2.13. Establishment of Lung Cancer Cells with C797S EGFR Mutation

H1975 lung cancer cells harboring the C797S EGFR mutation were established using retroviral transduction of the *EGFR* gene with T790M and C797S mutations. Briefly, the C797S mutation was generated by site-directed mutagenesis from pBabe EGFR (L858R/T790M) plasmid (Addgene, Cambridge, MA, USA) using a QuikChange™ Site-Directed Mutagenesis kit (Stratagene California, San Diego, CA, USA) according to the manufacturer’s protocol. The following primers were used for site-directed mutagenesis: forward, 5′-CAGCTCATGCCCTTCGGCAGCCTCCTGGACTATGTCCGGG-3′, and reverse, 5′-CCCGGACATAGTCCAGGAGGCTGCCGAAGGGCATGAGCTG-3′. The resultant pBabe EGFR (L858R/T790M/C797S) plasmid was then transfected into HEK 293 Phoenix ampho packaging cells (ATCC, Manassas, VA) by Fu-GENE6 transfection reagent (Roche, Lewes, UK). Forty-eight hours after transfection, the supernatant was collected for transduction of the retrovirus into the H1975 lung cancer cells. The lung cancer cells were selected with puromycin for 3 weeks, and then the remaining cells were amplified for further analysis.

### 2.14. Semi-Quantitative Reverse Transcription Polymerase Chain Reaction (RT-PCR) for EGFR

Total RNA was extracted from the H1975 lung cancer cells using an RNeasy Mini Kit (QIAGEN, Hilden, Germany) according to the manufacturer’s instructions, and then cDNA was synthesized using an iScript^™^ cDNA Synthesis Kit (Bio-Rad Laboratories, Munich, Germany). The following primers were used: EGFR (L858R/T790M/C797S), forward, 5′-CTTTATCCAGCCCTCAC-3′, reverse, 5′- TGTTACTCGTGCCTTGGC-3′, and β-Actin, forward, 5′-CCTGGACTTCGAGCAAGAGATG-3′, reverse, 5′-AGGAAGGAAGGCTGGAAGAGTG-3′. The following conditions were used for RT-PCR: 95 °C for 2 min, 35 cycles of 95 °C for 15 s, 50 °C for 15 s, 72 °C for 30 s and 72 °C for 5 min. The product was then stored at 4 °C.

## 3. Statistical Analysis

A Student’s *t*-test was used to compare the results between the two different groups of samples; a one-way analysis of variance (ANOVA) with Tukey’s post hoc test was used for more than three groups; and a two-way ANOVA Bonferroni’s post hoc test was used for the mouse xenograft results. The GraphPad Prism 5 software (GraphPad Software, Inc., San Diego, CA, USA) was used for statistical analysis. Significance was considered as *p* < 0.05 with two-sided analysis.

## 4. Results

### 4.1. Overexpression of ANXA1 in Lung Cancer Cells

**Figure 1:**
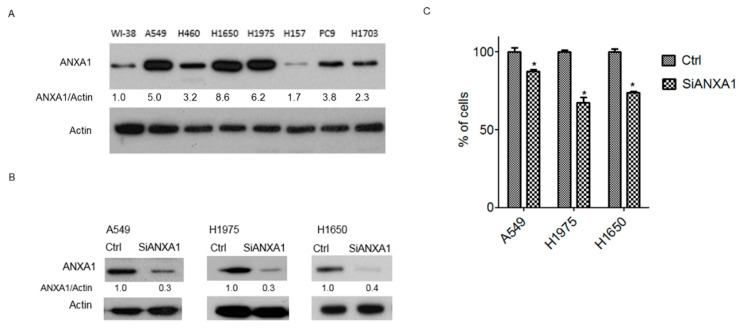
(**A**) Western blot analysis of ANXA1 in lung cancer cells. Internal control: actin. WI-38: human lung fibroblast; A549: adenocarcinoma; H460: large cell carcinoma; H1650: adenocarcinoma; EGFR mutation: exon 19 deletion; H1975: adenocarcinoma; EGFR mutation: L858R + T790M; H157: squamous cell carcinoma; PC9: adenocarcinoma; EGFR: exon 19 deletion; H1703: adenocarcinoma. (**B**) Western blot analysis of ANXA1 in A549 and H1975 lung cancer cells. The cells were transfected with 50 nM scrambled control (Ctrl) and ANXA1 (SiANXA1) siRNA for 72 h. Actin was used for internal control. The relative expression of ANXA1 was normalized to actin using Ctrl group as the control. (**C**) Cell numbers were determined in A549, H1975 and H1650 lung cancer cells after transfection of 50 nM scrambled control (Ctrl) and ANXA1 (SiANXA1) siRNA for 48 h. Cell numbers were normalized to control siRNA transfected groups. The experiments were performed in triplicate. “*” denotes *p* < 0.05. Uncropped western blot figures were included in Supplementary Materials.

We examined the expression of ANXA1 in NSCLC lung cancer cells using Western blot analysis. The results showed that among the six lung cancer cell lines, all cell lines (A549, H460, H1650, H1975, H157, PC9 and H1703) expressed ANXA1 at levels substantially higher than those expressed by normal human fetal lung fibroblast cells (WI-38) (Figure 1A). The overexpression of ANXA1 was observed in H1650 and H1975 lung adenocarcinoma cells with *EGFR* mutations (H1650: exon 19 deletion; H1975: L858R + T790M) and A549 lung adenocarcinoma cells wild-type *EGFR* gene.

### 4.2. Knockdown of ANXA1 Inhibits the Growth of Lung Cancer Cells

A siRNA knockdown study was performed to observe the effect of ANXA1 on lung cancer cells with an overexpression of ANXA1. The expression of ANXA1 was downregulated by ANXA1 siRNA in A549, H1975 and H1650 lung cancer cells (Figure 1B). After ANXA1 knockdown, a significantly decreased growth was observed in the lung cancer cells studied (Figure 1C).

### 4.3. Knockdown of ANXA1 Enhanced Osimertinib Chemosensitivity and Inhibited Tumorigenesis in Lung Cancer Cells with EGFR Mutations

**Figure 2:**
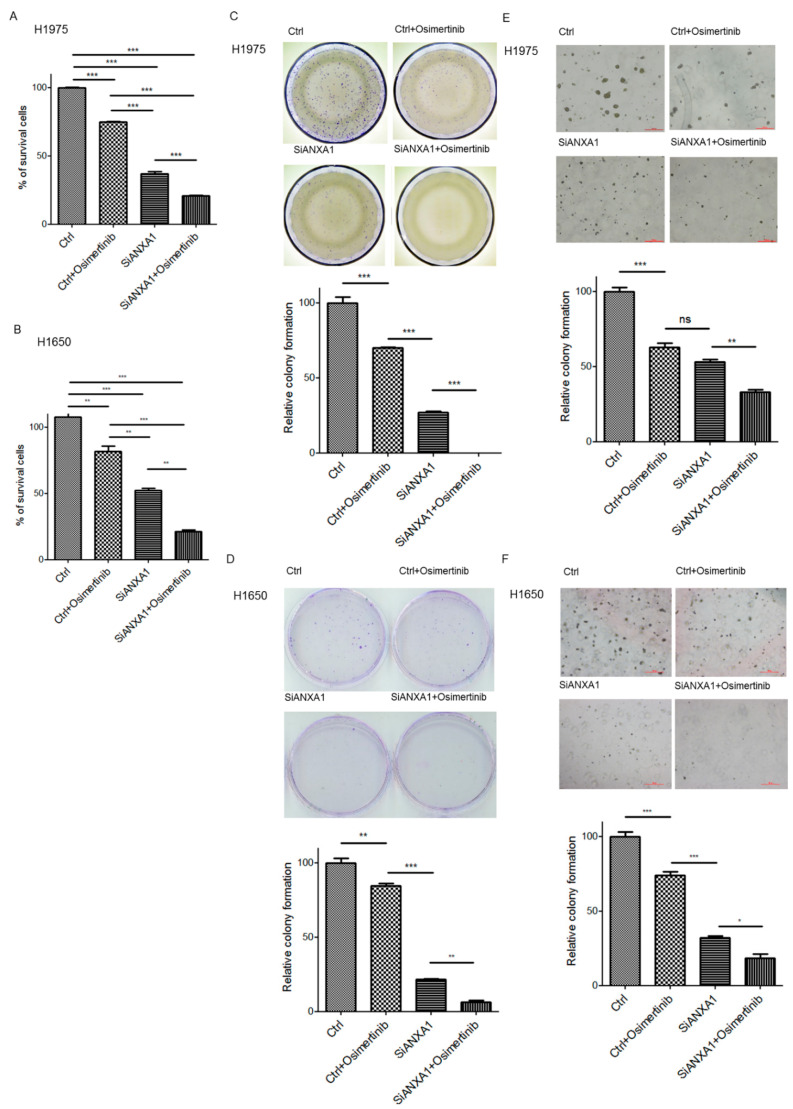
Cell numbers were determined in (**A**) H1975 and (**B**) H1650 lung cancer cells after transfection of 50 nM scrambled control (Ctrl) and ANXA1 (SiANXA1) siRNA with or without 10 nM Osimertinib for 72 h. Cell numbers were normalized to control siRNA transfected groups. The experiments were performed in triplicate. Anchorage-dependent colony formation assay in (**C**) H1975 and (**D**) H1650 lung cancer cells. After transfection of 50 nM scrambled control (Ctrl) and ANXA1 (SiANXA1) siRNA, cells were treated with or without 10 nM Osimertinib for 14 days. Anchorage-independent 3D culture in (**E**) H1975 and (**F**) H1650 lung cancer cells after transfection of 50 nM scrambled control (Ctrl) and ANXA1 (SiANXA1) siRNA with or without 10 nM Osimertinib for 10 days. Relative colony formation is represented by normalization of the colony number to the control group and shown as bar ± standard deviation in triplicate experiments. “*ns*” denotes nonspecific. “*” denotes *p* < 0.05; “**” denotes *p* < 0.01; “***” denotes *p* < 0.001.

The effect of ANXA1 on chemosensitivity and tumorigenesis to Osimertinib was studied. In H1975 and H1650 lung cancer cells with EGFR mutations, the combination treatment of ANXA1 siRNA and Osimertinib resulted in significantly decreased survival cells compared to the Osimertinib group (Figure 2A,B). In the anchorage-dependent colony formation assay, an increased growth inhibition of Osimertinib was also observed in the ANXA1 knockdown H1975 and H1650 lung cancer cells (Figure 2C,D). In the anchorage-independent soft agar colony formation assay, an increased growth inhibition of Osimertinib was also observed in the ANXA1 knockdown H1975 and H1650 lung cancer cells (Figure 2E,F).

### 4.4. Knockdown of ANXA1 Inhibited Invasion and Migration in Lung Cancer Cells with EGFR Mutations

**Figure 3:**
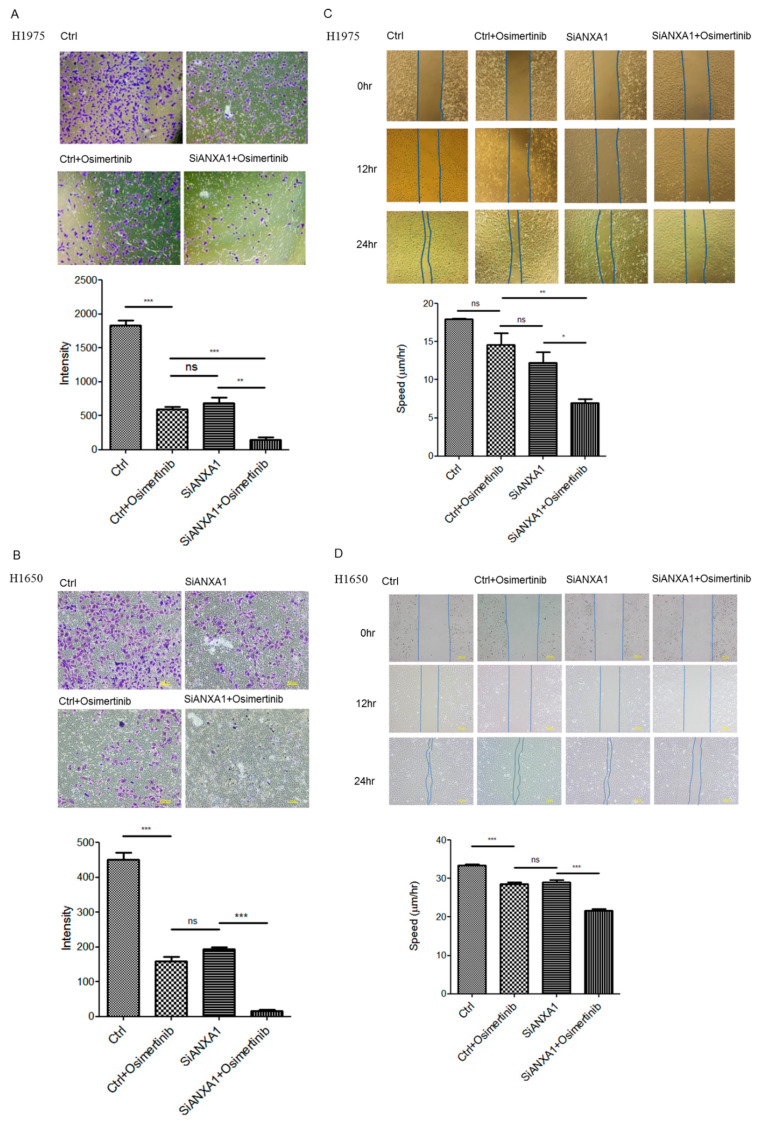
Invasion assay in (**A**) H1975 and (**B**) H1650 lung cancer cells after transfection of 50 nM scrambled control (Ctrl) and ANXA1 (SiANXA1) siRNA with or without 10 nM Osimertinib for 72 h. Intensity of cells is represented as bar ± standard deviation in triplicate experiments. Wound healing assay in (**C**) H1975 and (**D**) H1650 lung cancer cells after transfection of 50 nM scrambled control (Ctrl) and ANXA1 (SiANXA1) siRNA with or without 10 nM Osimertinib for 12 and 24 h. The speed of cellular migration at 24 h is represented as bar ± standard deviation in triplicate experiments. “*ns*” denotes nonspecific. “*” denotes *p* < 0.05; “**” denotes *p* < 0.01; “***” denotes *p* < 0.001.

The trans-well invasion assay and wound healing assay were performed to study the effect of ANXA1 knockdown and Osimertinib on the ability for invasion and metastasis in lung cancer cells. In the invasion assay, an increased invasion inhibition of Osimertinib was also observed in the ANXA1 knockdown H1975 and H1650 lung cancer cells (Figure 3A,B). In the wound healing assay, the migration of H1975 lung cancer cells was significantly inhibited in the Osimertinib treatment and ANXA1 knockdown group (Figure 3C,D).

### 4.5. Knockdown of ANXA1 with Osimertinib Inhibited EGFR Down-Stream Pathways and Increased Apoptosis in Lung Cancer Cells with EGFR Mutations

**Figure 4:**
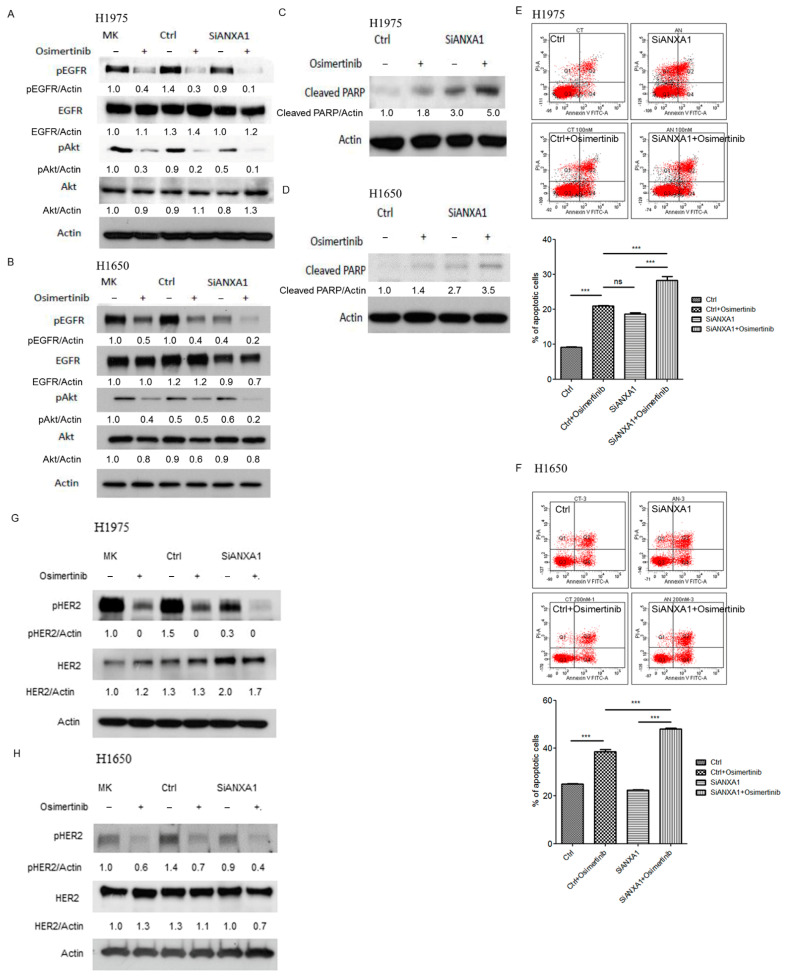
Western blot analysis of EGFR, pEGFR (Tyr1068), Akt (Ser473), pAkt and actin in (**A**) H1975 and (**B**) H1650 lung cancer cells transfected with 50 nM scrambled control (Ctrl) and ANXA1 (SiANXA1) siRNA for 72 h and then treated with 10 nM Osimertinib for 3 h. MK: mock, without transfection of siRNA. The relative expression of pEGFR, EGFR, pAkt and cleaved PARP were normalized to actin using MK group as the control. MK: mock; pEGFR: phosphor EGFR; pAkt: phosphoAkt. Western blot analysis of cleaved PARP in (**C**) H1975 and (**D**) H1650 lung cancer cells transfected with 50 nM scrambled control (Ctrl) and ANXA1 (SiANXA1) siRNA for 72 h and then treated with or without 100 nM Osimertinib for 24 h. Actin was used for internal control. The relative expression of ANXA1 was normalized to actin using Ctrl group as the control. Apoptosis assay by Annexin V-FITC in (**E**) H1975 and (**F**) H1650 lung cancer cells after transfection of 50 nM scrambled control (Ctrl) or ANXA1 (SiANXA1) siRNA with or without 100 nM Osimertinib for 24 h. The percentage of apoptotic cells is represented as bar ± standard deviation in triplicate experiments. Western blot analysis of HER2, pHER2 (Tyr877) and actin in (**G**) H1975 and (**H**) H1650 lung cancer cells transfected with 50 nM scrambled control (Ctrl) and ANXA1 (SiANXA1) siRNA for 72 h and then treated with 10 nM Osimertinib for 3 h. The relative expression of pHER2 and HER2 were normalized to actin using MK group as the control. MK: mock; pHER2: phosphor HER2. “*ns*” denotes nonspecific. “***” denotes *p* < 0.001. Uncropped western blot figures were included in Supplementary Materials.

The changes in the down-stream signaling pathways of EGFR in lung cancer cells with and without ANXA1 knockdown or Osimertinib treatment were then studied. A decreased phosphorylation of EGFR (Tyr1068) and the down-stream Akt (Ser473) pathway was noted in ANXA1 knockdown with Osimertinib treatment H1975 and H1650 lung cancer cells (Figure 4A,B). Further studies showed that an increased cleaved PARP was noted in ANXA1 knockdown with Osimertinib-treated lung cancer cells (Figure 4C,D). An increased apoptotic cell was observed in ANXA1 knockdown with Osimertinib treatment lung cancer cells (Figure 4E,F). The phosphorylation of HER2 (Tyr877) was also evaluated in H1975 and H1650 lung cancer cells. A decreased phosphorylation of HER2 was observed in H1975 and H1650 lung cancer cells after the knockdown of ANXA1 and Osimertinib treatment (Figure 4G,H).

### 4.6. Knockdown of ANXA1 with Osimertinib Inhibited Tumor Growth in the Mice Xenograft Model of Lung Cancer Cells with EGFR Mutations

**Figure 5:**
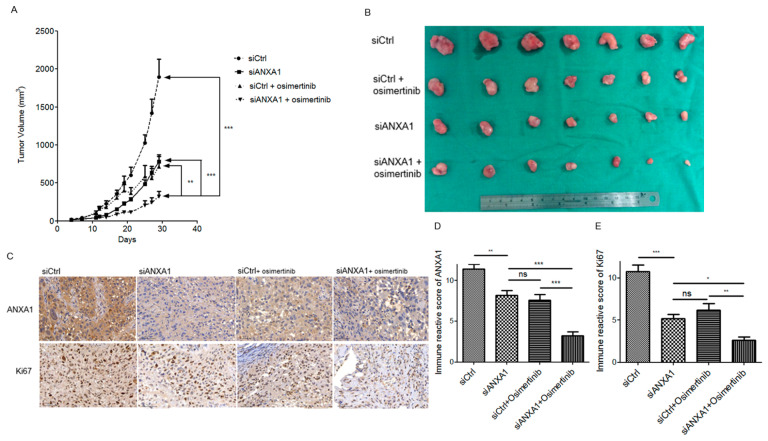
(**A**) Nude mice xenograft model of the H1975 lung cancer cells. The mice were injected intratumorally with non-target siRNA or ANXA1 siRNA with or without parenteral Osimertinib. The data were represented as mean tumor volume ± standard deviation (*n* = 7 in each group). (**B**) Tumors of the H1975 xenograft model. (**C**) Representative graph of the ANXA1 and Ki67 IHC staining. Magnification: 200X. Scale bar: 20 μM. (**D**) Immune reactive score (IRS) of ANXA1. E. IRS of Ki67. The IRS data were represented as mean ± standard deviation (*n* = 5 in each group). siCtrl: nontarget siRNA. siANXA1: ANXA1 siRNA. “*ns*” denotes nonspecific. “*” denotes *p* < 0.05; “**” denotes *p* < 0.01; “***” denotes *p* < 0.001.

A mice xenograft model was established using H1975 lung cancer cells. Non-target and ANXA1 siRNAs were injected into xenografts tumors. The mice were treated with or without Osimertinib. A significantly decreased tumor volume was observed in the group of mice treated with ANXA1 siRNA and Osimertinib (Figure 5A,B). Further IHC staining showed a decreased expression of ANXA1 (Figure 5C,D) and Ki-67 (Figure 5C,E) in the tumors treated with ANXA1 siRNA and Osimertinib.

### 4.7. Overexpression of ANXA1 Decreased Chemosensitivity to Osimertinib

**Figure 6:**
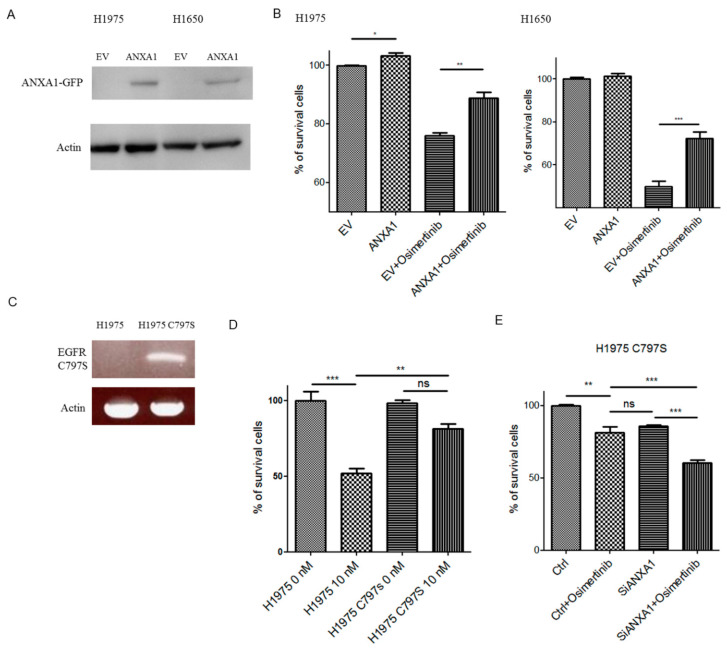
(**A**). Western blot analysis of ANXA1-GFP in H1975 and H1650 lung cancer cells transfected with pCMV6-AC-GFP (EV) and pCMV6-ANXA1-GFP (ANXA1). Cell numbers were determined in (**B**) H1975 and H1650 lung cancer cells treated with or without 10 (H1975) or 20 nM (H1650) of Osimertinib for 72 h. Cell numbers were normalized to the EV group. The experiments were performed in triplicate. (**C**) RT-PCR of the ectopic EGFR in H1975 lung cancer cells transfected with L858R + C797S EGFR mutations (H1975C797S). (**D**) Cell numbers were determined in H1975 and H1975C797S lung cancer cells treated with or without 10 nM Osimertinib for 72 h. Cell numbers were normalized to the H1975 group. The experiments were performed in triplicate. (**E**) H1975C797S lung cancer cells after transfection of 50 nM scrambled control (Ctrl) and ANXA1 (SiANXA1) siRNA with or without 10 nM Osimertinib for 72 h. Cell numbers were normalized to control siRNA transfected groups. The experiments were performed in triplicate. “*ns*” denotes nonspecific. “*” denotes *p* < 0.05; “**” denotes *p* < 0.01; “***” denotes *p* < 0.001. Uncropped western blot figures were included in Supplementary Materials.

ANXA1 was expressed in H1975 and H1650 lung cancer cells (Figure 6A). An increased growth (Figure 6B, H1975) and decreased chemosensitivity to Osimertinib (Figure 6B) were observed in the ectopic ANXA1 overexpressed H1975 and H1650 lung cancer cells.

### 4.8. Knockdown of ANXA1 Enhanced Osimertinib Chemosensitivity in Osimertinib-Resistant C797S Lung Cancer Cells

Osimertinib-resistant H1975 stable lung cancer cells with the C797S mutation (H1975 C797S) were established using a retroviral transduction of the *EGFR* gene with L858R and C797S mutations (Figure 6C). A decreased chemosensitivity to Osimertinib was observed in the H1975 C797S lung cancer cells (Figure 6D). The knockdown of ANXA1 also increased the chemosensitivity to Osimertinib in the H1975 C797S lung cancer cells (Figure 6E).

## 5. Discussion

Similar to a previous study [12], our study further confirmed that ANXA1 is overexpressed in lung cancer cells with or without EGFR mutations. Since the knockdown of ANXA1 is associated with the decreased growth, invasion and migration of lung cancer cells, ANXA1 may play the role of a tumor promoter in lung cancer cells. Our study results also showed an increased inhibitory effect on the growth of lung cancer cells of Osimertinib in ANXA1 knockdown H1975 and H1650 lung cancer cells. Furthermore, the overexpression of ANXA1 decreased chemosensitivity to Osimertinib in lung cancer cells and the knockdown of ANXA1 increased chemosensitivity to Osimertinib in lung cancer cells with the Osimertinib-resistant C797S mutation. Therefore, ANXA1 may be an attractive target for lung cancer therapy and to enhance the treatment effects of Osimertinib on lung cancer cells with EGFR mutations.

Our study results imply that the expression of ANXA1 is associated with an important role in the tumorigenesis of lung cancer. Further study showed that a possible mechanism is that the knockdown of ANXA1 attenuates the phosphorylation of EGFR and its down-stream Akt pathway in combination with Osimertinib. In addition, the knockdown of ANXA1 also increased apoptosis in combination with Osimertinib. For the first time, we observed that the knockdown of ANXA1 may potentiate the effect of EGFR- TKI. The development of acquired resistance after Osimertinib treatment in T790M positive lung cancer cells may be due to several mechanisms. The EGFR C797S mutation in exon 20, which affects the binding site of Osimertinib, accounts for 31% of acquired resistance after Osimertinib treatment [17]. In our study, the knockdown of ANXA1 also increased chemosensitivity to Osimertinib in T790M positive lung cancer cells expression stably expressing L858R + C797S EGFR mutations (H1975C797S). Our efforts may help in the development of the treatment strategies to improve the effects of EGFR-TKI drugs in the future.

The knockdown of ANXA1 was also associated with the decreased migration and invasion of lung cancer cells in our study as well as another study [12]. Several mechanisms have been observed regarding the role of ANXA1 in cancer metastasis. ANXA1 promotes invasion and migration through the TGF-beta/Smad signaling pathway and actin reorganization [18]. ANXA1 acts as an extracellular similar to the formyl peptide receptor (FPR) ligand or cytoskeleton remodeling factor and promotes the migration and invasion of cancer cells [19,20]. ANXA1 promotes metastasis via the stimulation of FPR and matrix metalloproteinase 2 expression [21]. ANXA1 also promotes the proliferation and metastasis of cancer cells through the binding of EphA2, the suppression of autophagy and the activation of the PI3K/AKT pathway [22,23] and promotes proliferation and migration via the regulation of the IL-6/JAK2/STAT3 pathway [24]. The EGFR pathway has been associated with the proliferation, metastasis and survival of cancer cells [25]. The association between ANXA1 and EGFR in lung cancer cells was observed in our study, and our study results may provide another mechanism in which ANXA1 promotes the metastasis of lung cancer cells through the modulation of the EGFR pathway. Osimertinib was reported to inhibit HER2 phosphorylation in lung cancer cells [26]. The association of the ANXA1and HER2 pathway is less clear. In our study, a decreased HER2 phosphorylation was observed in H1975 and H1650 lung cancer cells after the knockdown of ANXA1 and Osimertinib treatment. Since HER2 amplification is the second common cause of acquired resistance to Osimertinib in T790M positive lung cancer [27], targeting ANXA1 may help to overcome acquired resistance through the inhibition of HER2 phosphorylation. However, further studies to elucidate the association of ANXA1and HER2 are warranted.

The research evidence showed that the expression of ANXA1 is conflicting between different cancer types and ANXA has tumor suppressor and promoter roles in different types of cancers [28]. For example, ANXA1 has tumor suppressor roles in prostate, oral squamous cell [29] and head and neck cancer [30]. On the contrary, ANXA1 has tumor promoter roles in pancreatic cancer [31], breast cancer [32], liver cancer [33], lung cancer [34], thyroid cancer [24] and esophageal cancer [35]. Our study results add another piece of evidence that ANXA1 plays various roles in different cancer types and its complex regulation mechanisms. More studies are still needed to further elucidate the role of ANXA1 as a diagnostic, predictive and therapeutic marker in lung cancer and other cancers.

In this study, a murine xenograft model was used to evaluate the effects of the combination of ANXA1 siRNA with Osimertinib in vivo using an Accell ANXA1 siRNA transfection developed by Daharmacon [36]. Through the unique modification of the siRNA structure and a proprietary sequence design algorithm to reduce off-target effects, the Accel siRNA could be used to transfect transfection-resistant cells [36,37] and has been reported to silence genes in several organs [38] and tissues [39] without transfection reagents. In our study, we further showed the successful knockdown of ANXA1 using Accell siRNA in the tumor xenograft animal model. The treatment value of ANXA1 siRNA in the augmentation of the treatment effects of Osimertinib was also proven, and anti-ANXA1 therapy may be applied in the future as an adjuvant strategy to EGFR-TKI therapy. Since drugs that are targeted for ANXA1 are currently unavailable, siRNA can be a new class of pharmaceutical drugs. Therapy based on siRNA may provide a powerful tool to identify specific and potent inhibitors of disease targets. The broad potential application of siRNA therapeutics has been proven by studies of animal models of human diseases [40] as well as in clinical use [41].

## 6. Conclusions

In conclusion, our study showed that ANXA1 is a candidate for target therapy toward NSCLC and targeting ANXA1 may enhance the effects of EGFR-TKI in lung cancer cells with EGFR mutations.

## Data Availability

All data are included in the article.

## Supplementary Materials

The following are available online at https://www.mdpi.com/article/10.3390/cancers13164106/s1, Uncropped western blot figures were included in Supplementary Materials.
