# Supplementary material for: The Association of Annexin A1 and Chemosensitivity to Osimertinib in Lung Cancer Cells
Source: Cancers (Basel). 2021 Aug 15;13(16):4106. doi: 10.3390/cancers13164106 (PMC8394458; doi:10.3390/cancers13164106)
Supplement: Supplementary file 1 [file cancers-13-04106-s001.zip › cancers-1250936-supplementary.pdf]

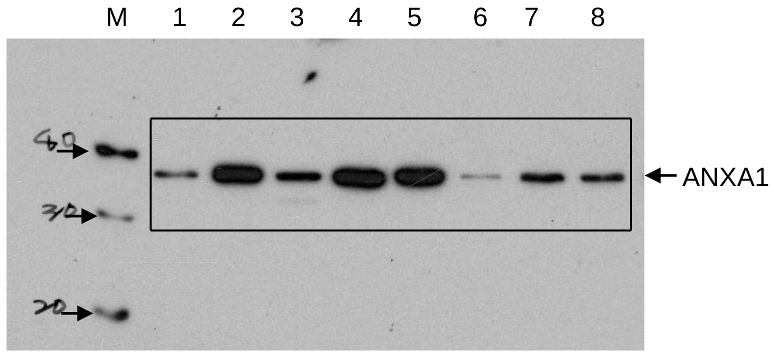

1.WI-38
2.A549
3.H460
4.H1650
5.H1975
6.H157
7.PC9
8.H1703

FIG 1A-1

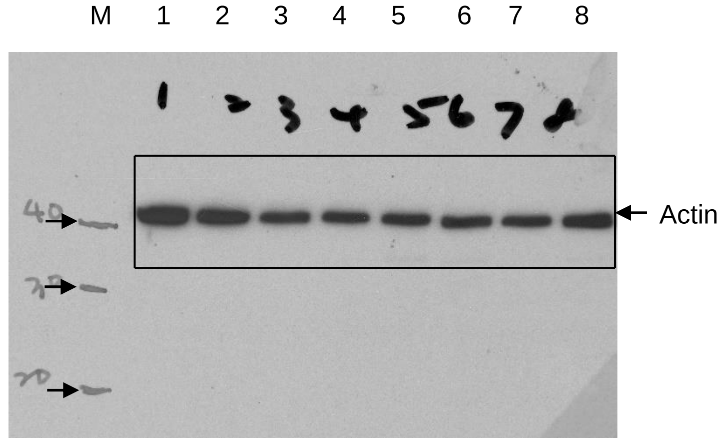

FIG-1A-2

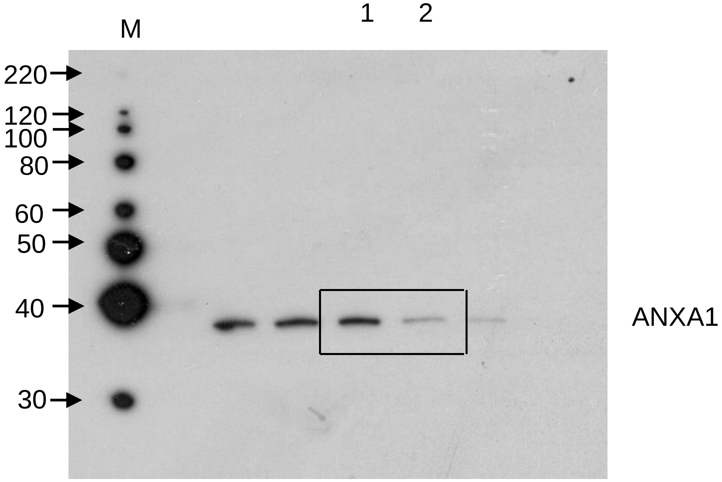

FIG 1B-1

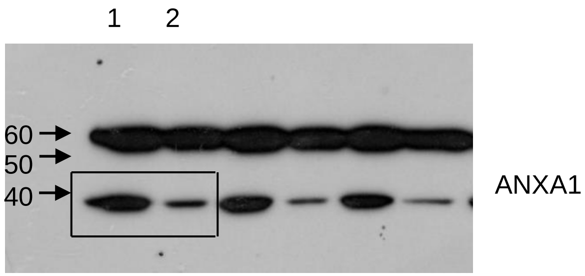

FIG 1B-2

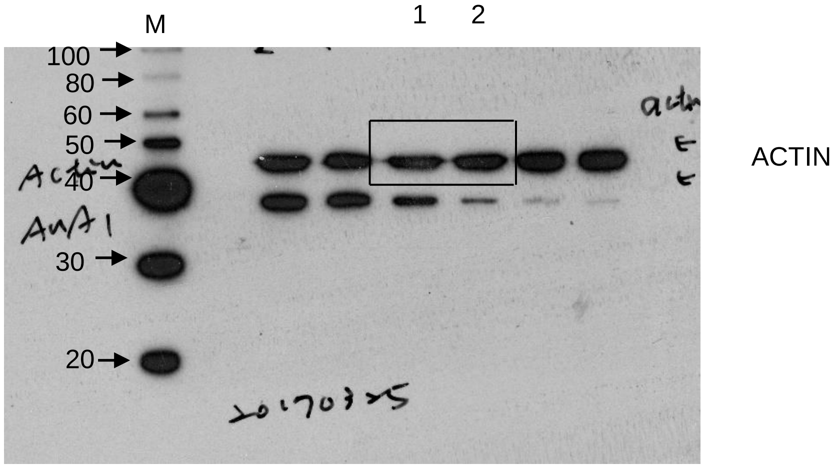

FIG 1B-1

1.A549 Ctrl

2.A549 ANXA1

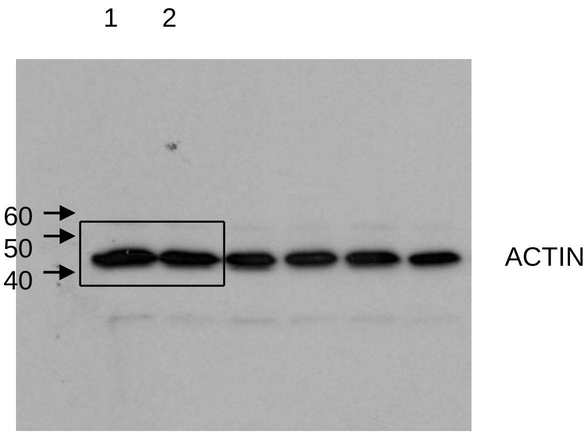

FIG 1B-2

1.H1975 Ctrl

2.H1975 ANXA1

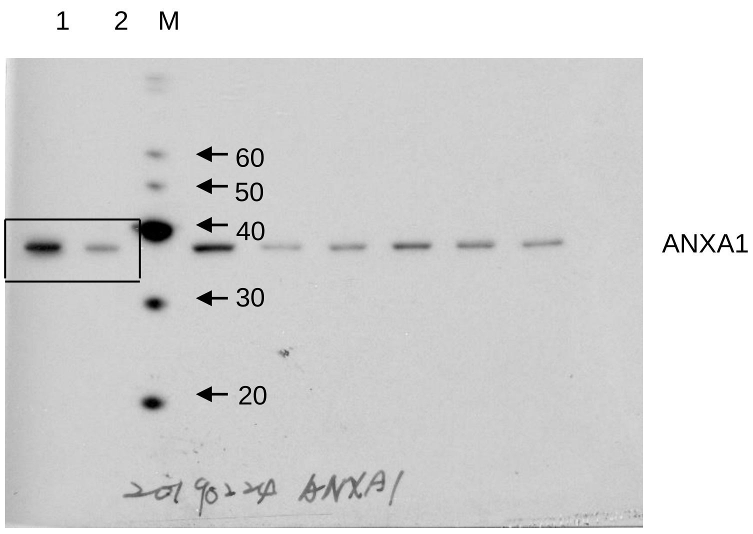

FIG 1B-3

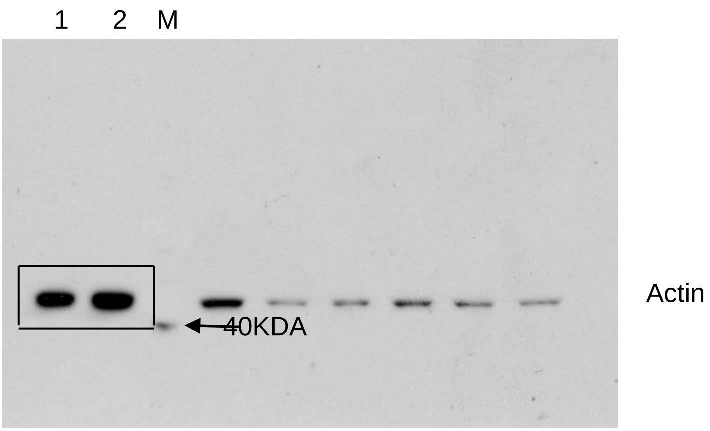

FIG 1B-3

1.H1650 Ctrl

2.H1650 ANXA1

H1975

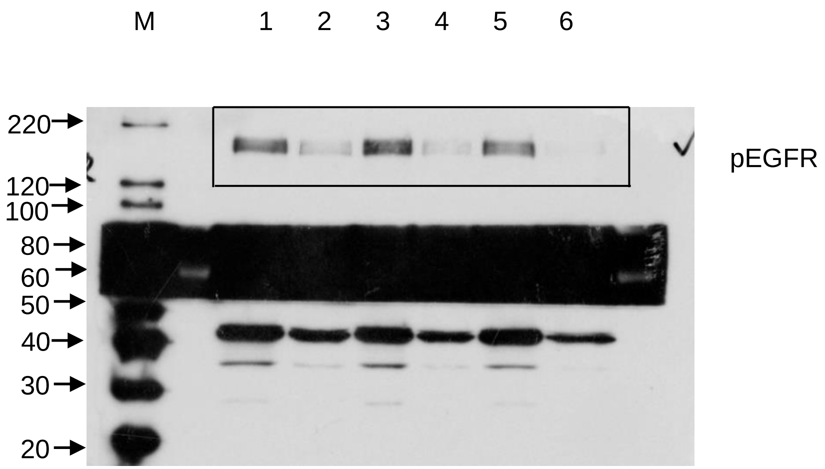

FIG-4A-1

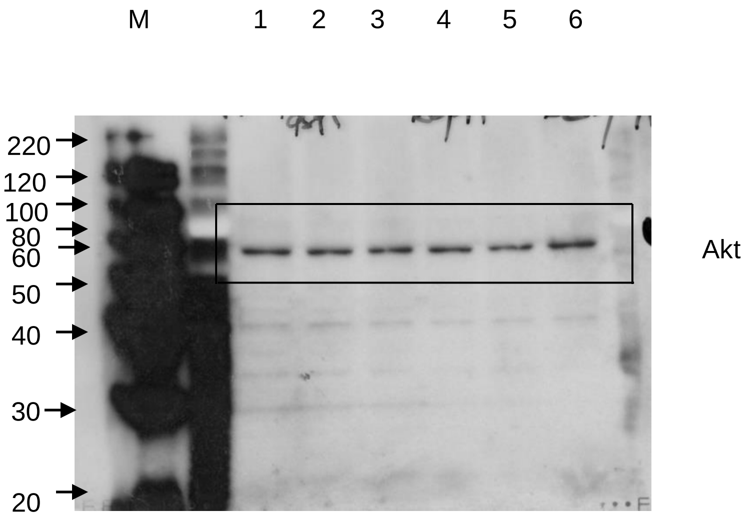

FIG-4A-4

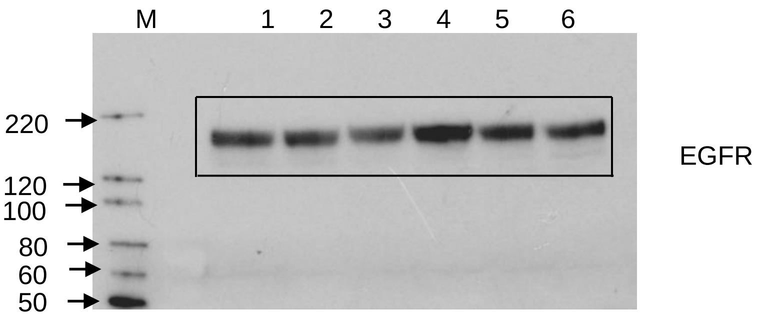

FIG-4A-2

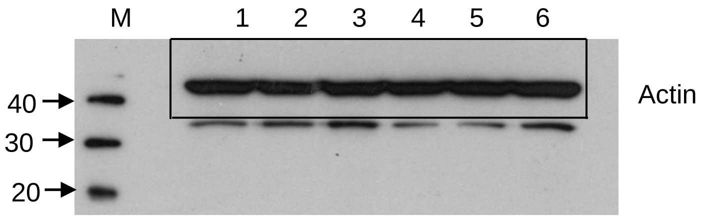

FIG-4A-5

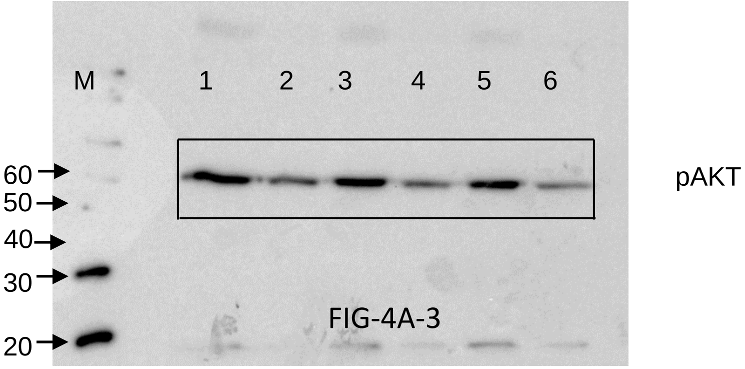

FIG-4A-3

1. Mock
2. Mock+Osimertinib
3. Ctrl
4. Ctrl+Osimertinib
5. SiANXA1
6. SiANXA1+Osimertinib

H1650

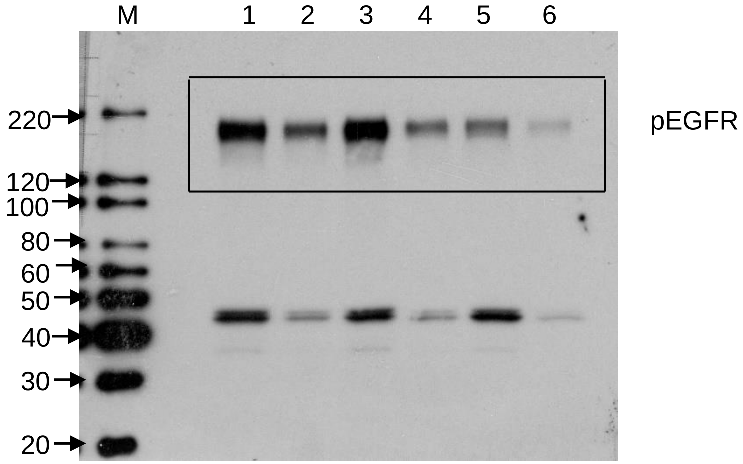

FIG-4B-1

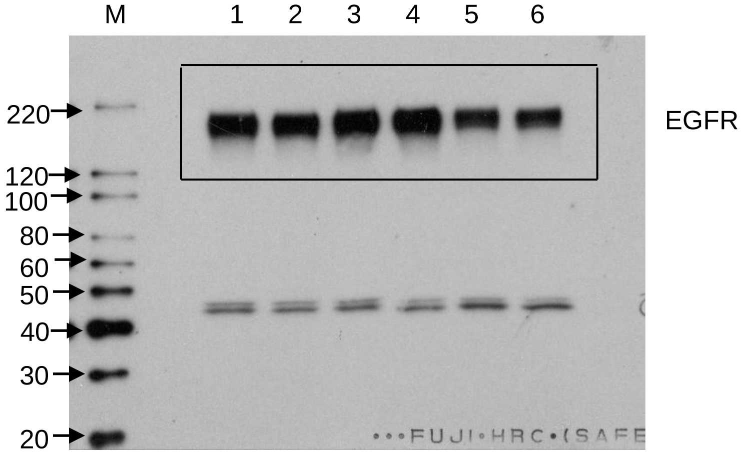

FIG-4B-2

1. Mock
2. Mock+Osimertinib
3. Ctrl
4. Ctrl+Osimertinib
5. SiANXA1
6. SiANXA1+Osimertinib

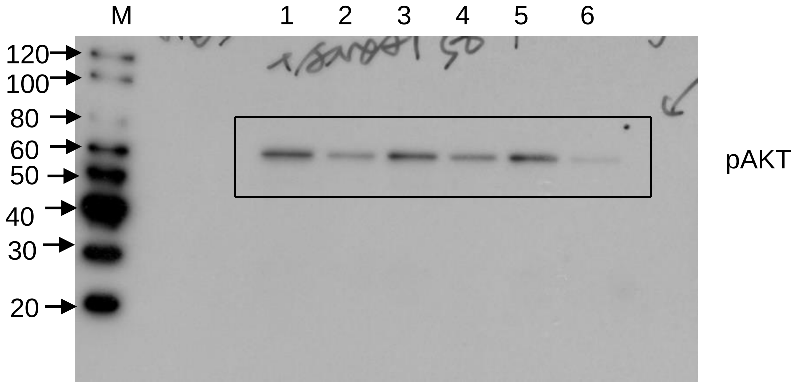

FIG-4B-3

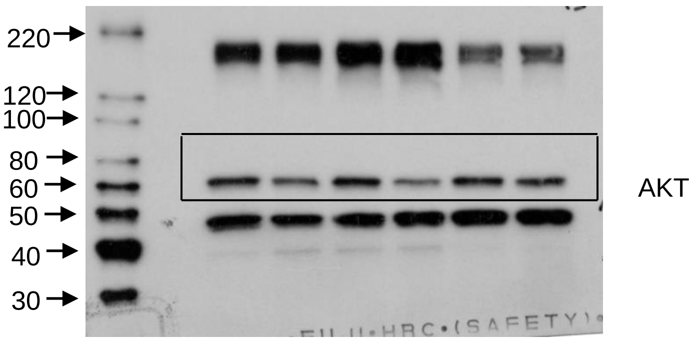

FIG-4B-4

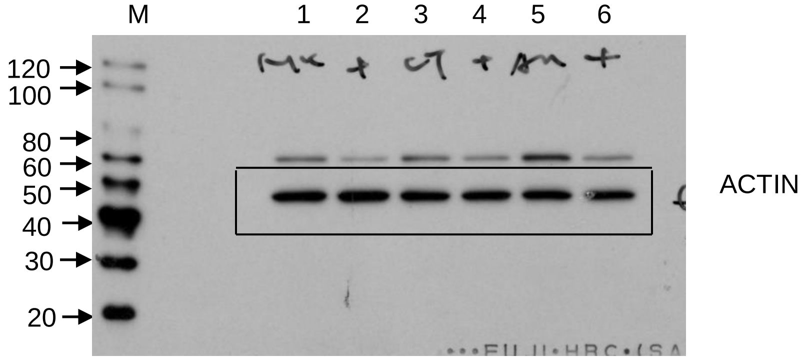

FIG-4B-5

H1975

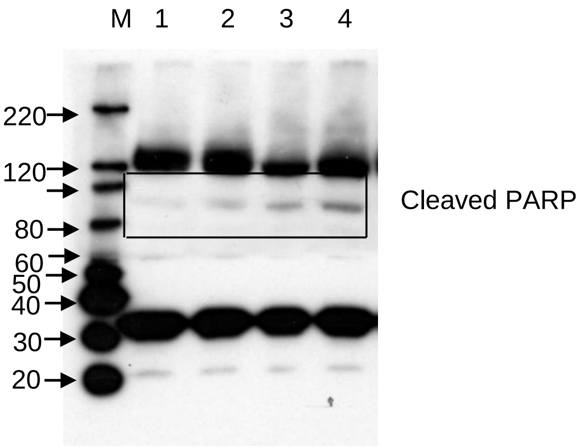

FIG-4C-1

H1650

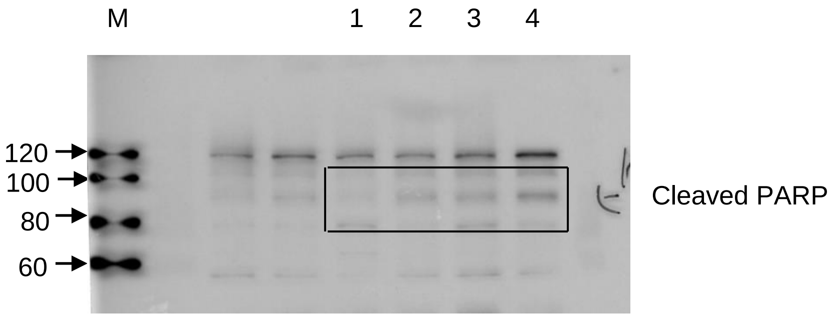

FIG-4D-1

H1975

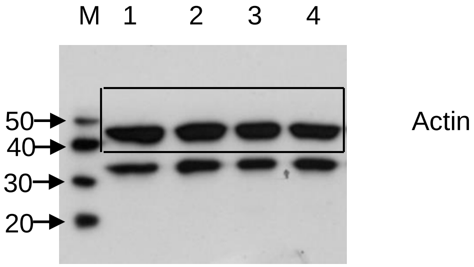

FIG-4C-1

H1650

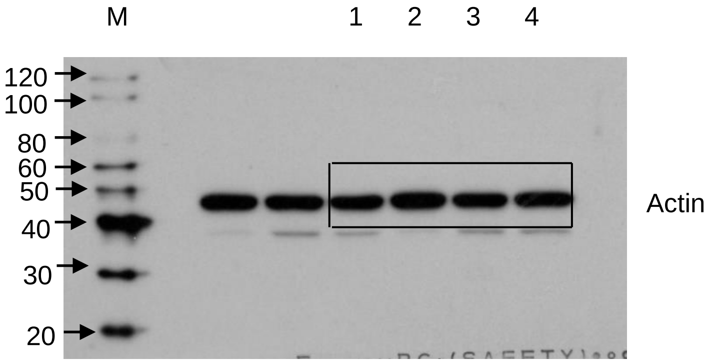

FIG-4D-1

1. Ctrl
2. Ctrl+Osimertinib
3. SiANXA1
4. SiANXA1+Osimertinib

H1975

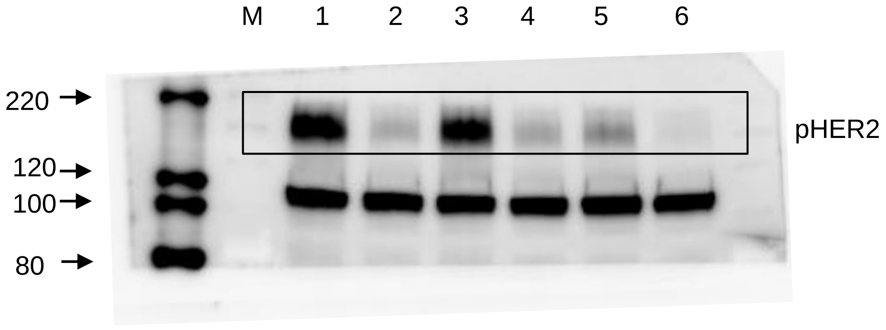

FIG-4G-1

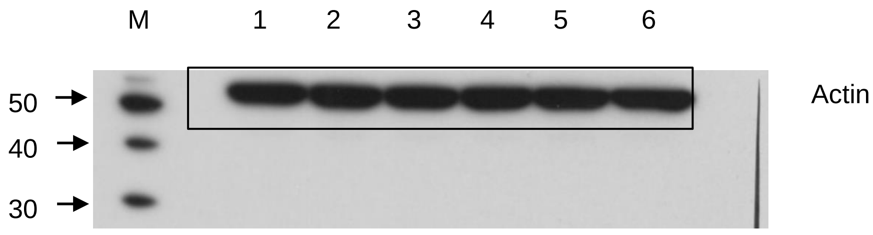

FIG-4G-3

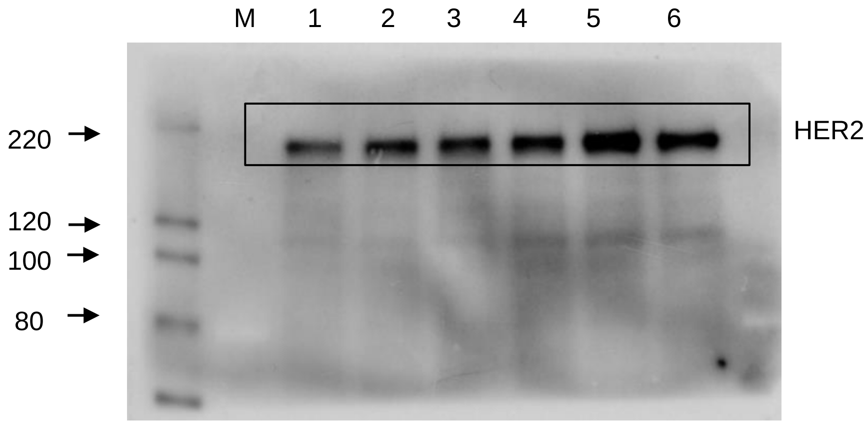

FIG-4G-2

1. Mock
2. Mock+Osimertinib
3. Ctrl
4. Ctrl+Osimertinib
5. SiANXA1
6. SiANXA1+Osimertinib

H1650

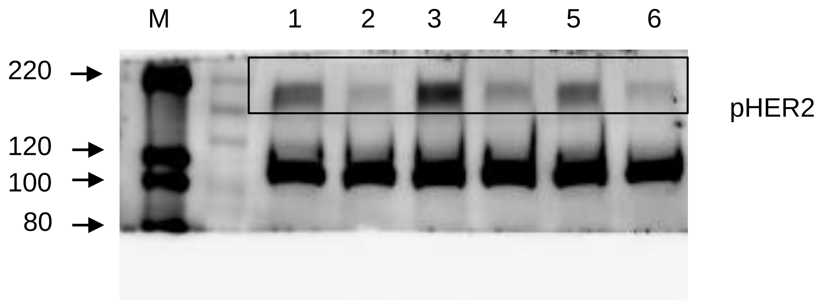

FIG-4H-1

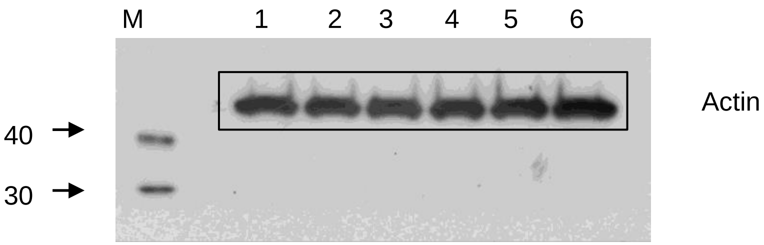

FIG-4G-3

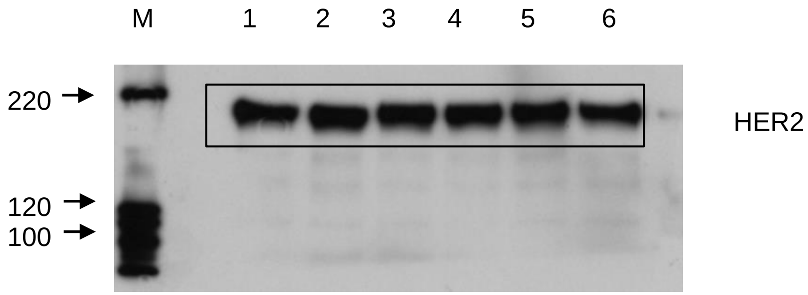

1. Mock
2. Mock+Osimertinib
3. Ctrl
4. Ctrl+Osimertinib
5. SiANXA1
6. SiANXA1+Osimertinib

FIG-4H-2

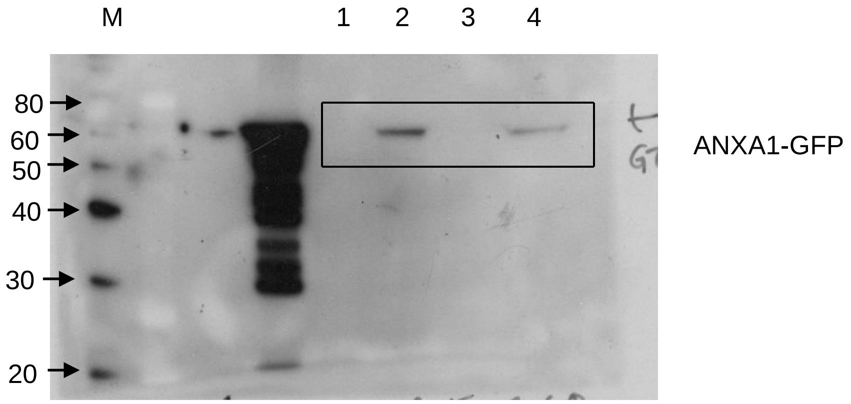

1.H1975 pCMV-6-AC-GFP(EV)

2.H1975 pCMV-6-ANXA1-GFP(ANXA1)

3.H1650 pCMV-6-AC-GFP(EV)

4.H1650 pCMV-6-ANXA1-GFP(ANXA1)

FIG.6A-1

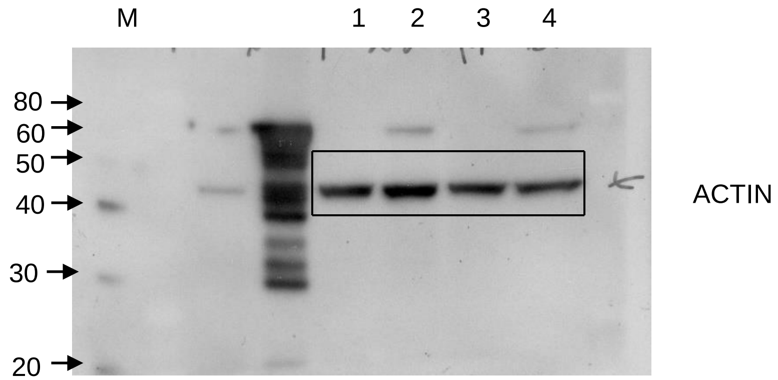

FIG.6A-2
